# Association between sanitary toilets and health poverty vulnerability among rural western Chinese adults aged 45 years and older: A cross-sectional study

**DOI:** 10.1371/journal.pone.0308688

**Published:** 2024-09-20

**Authors:** Ximin Ma, Qi Hu, Jiahui He, Chunsheng Li, Kexin Chen, Wenlong Wang, Hui Qiao

**Affiliations:** 1 School of Public Health, Ningxia Medical University, Yinchuan, China; 2 Key Laboratory of Environmental Factors and Chronic Disease Control, Yinchuan, China; 3 School of Humanities and Management, Ningxia Medical University, Yinchuan, China; Cranfield University, UNITED KINGDOM OF GREAT BRITAIN AND NORTHERN IRELAND

## Abstract

This study aimed to investigate the association between sanitary toilets and health poverty vulnerability among rural western Chinese adults aged 45 years and older. Using data from the ’Rural Household Health Inquiry Survey’ conducted in 2022, a three-stage feasible generalized least squares method was employed to calculate health poverty vulnerability. Propensity score matching (PSM) and mediation effect analysis were used to assess the association between sanitary toilets and health poverty vulnerability among rural western Chinese adults aged 45 years and older and the mechanisms underlying this impact. This study revealed that the use of sanitary toilets was significantly associated with decreased health poverty vulnerability in adults over 45 years of age. Heterogeneity analysis revealed that this effect was more pronounced among males (β = -0.0375, *P*<0.05), those aged 60–74 years (β = -0.0476, *P*<0.05), and households with middle income (β = -0.0590, *P*<0.01). Mediation effect analysis identified total household income (a×b = -0.0233, *P*<0.05), household size (a×b = -0.0181, *P*<0.01), number of household laborers (a×b = -0.0107, *P*<0.01), and registered poor households (a×b = -0.0081, *P*<0.01) as the mediating factors between sanitary toilets and health poverty vulnerability. The provision of sanitary toilets has been instrumental in mitigating health-related poverty among middle-aged and elderly people residing in rural areas. By improving household livelihood capital, the vulnerability of these individuals to health-related poverty can be significantly reduced.

## Introduction

Eliminating poverty is considered a significant challenge in the 21st century [1]. According to World Bank data from 2018, 736 million people worldwide live on less than $1.90 a day [2,3]. The first of the 17 Sustainable Development Goals set by the United Nations is to eradicate all forms of poverty worldwide by 2030 [4,5]. China, the country with the largest rural poor population globally, has a poverty issue that is of particular concern. Since its reform and opening up in 1978, China has made remarkable progress in poverty reduction. Data from the National Bureau of Statistics indicate that between 1978 and 2020, the number of rural poor people in China decreased from 770 million to 5.51 million, and the poverty rate decreased from 97.5% to 0.6% [6,7]. In 2015, it became the first developing country to achieve its Millennium Development Goals, contributing more than 70% to global poverty reduction [8].

Although China has eradicated absolute poverty, poverty is dynamically changing, and the current nonpoverty status does not guarantee a continuous future. Some nonpoor families or individuals may fall into poverty due to unemployment or health issues. According to the World Bank (2020), the COVID-19 pandemic is expected to significantly impact poverty through various channels, including health and income setbacks [9]. A recent study conducted in China revealed that 7.1% of families that have never been poor are expected to fall into poverty due to the pandemic [10]. The National Health Commission’s 2015 data revealed that illness affected 44.1% of the impoverished population, either causing poverty or leading to reimpoverishment [11,12].

As China’s population ages, the proportion of the elderly population continues to increase. By 2021, the population aged 65 and above will exceed 200 million, accounting for 14.2% of the population [13,14]. The deepening aging process has made the health of middle-aged and elderly adults a focal point of public health concern. Rural middle-aged and elderly adults, as vulnerable groups, have a lower economic status, limited sources of income, and a greater incidence of chronic diseases [15]. Compared with their urban counterparts, they are more susceptible to health issues [16]. This increases the risk of this group falling into poverty or returning to poverty because of health problems.

To consolidate the achievements of poverty alleviation and prevent middle-aged and elderly adults in rural China from falling back into poverty due to illness, it is imperative to conduct a forward-looking assessment of health-related poverty issues and analyze their main determinants. The World Bank first introduced the concept of poverty vulnerability," defining it as the probability of a household falling into poverty in the future [17]. The World Health Organization (WHO) emphasizes the importance of well-being: "Health is a state of complete physical, mental, and social well-being and not merely the absence of disease or infirmity [18]. Building on this, Liu Yue et al. [19] proposed the concept of "health poverty vulnerability," which is defined as the probability that, due to health-related risks, a family’s or individual’s welfare decreases below the poverty line, reflecting the risk of falling into poverty in the future. Health poverty vulnerability can serve as a predictive indicator and early warning signal for the risk of falling into poverty or returning to poverty due to illness [20].

Despite economic growth and improvements in living standards in China, the issue of health poverty has not been completely resolved. This problem not only harms the well-being of individuals and families but also hinders socioeconomic development. In the field of health poverty vulnerability research, scholars have explored aspects such as poverty alleviation policies, health shocks, social capital, and social support networks, with research subjects including the rural elderly and women of childbearing age [21,22]. In addition, scholars have studied the health poverty vulnerability of specific disease patient groups, such as those with chronic diseases and type 2 diabetes [23,24], and have conducted in-depth analyses of the impact of informal social support, health shocks, and social capital on the health poverty vulnerability of rural residents [17,25]. Regarding the relationship between toilet type and health poverty vulnerability, existing studies have found that toilet type is a key factor affecting health poverty vulnerability [20,26]. Some studies have emphasized strategies to alleviate health poverty vulnerability by improving and promoting health and have proposed potential intervention methods [27].

Since 2015, China has seen significant improvements in sanitation, particularly in terms of the prevalence of sanitary toilets [28]. By 2021, it is expected that the urban sanitary toilet penetration rate will reach 97.6%, whereas the rural rate will reach 82.6% [29]. However, there are significant imbalances in sanitary toilet access across different rural areas in China, especially in the rural areas of western China. A survey revealed that the sanitary toilet penetration rates in Guyuan city, Ningxia Hui Autonomous Region, were 39.81%, 29.90%, and 31.94% between 2018 and 2020 [30], respectively. To improve sanitation in rural areas, the Chinese government has launched a "toilet revolution" to provide rural residents with access to sanitary toilets [31]. This initiative is closely related to the Patriotic Sanitation Campaign, which began in the 1950s to improve hygiene and reduce the incidence of disease [32]. Since 2004, the Chinese government has allocated approximately 8.64 billion RMB for rural toilet renovation. The goal of the toilet revolution is to achieve 85% and 100% coverage of sanitary toilets by 2020 and 2030, respectively [33]. The hygiene level of toilets is closely related to residents’ health. Fecal contamination of food and water sources leads to intestinal diseases, resulting in the death of 1.5 million children under the age of five each year, a figure higher than the number of deaths caused by AIDS and malaria [34]. Epidemiological studies have shown that a lack of sanitary toilets can intensify the spread of pathogens, such as *E*. *coli* and Salmonella, thereby increasing the incidence rates of diseases, such as helminths, schistosomiasis, malaria, and diarrhea [35]. In addition, the frequency of diseases caused by fecal pathogens is closely related to malnutrition, growth stunting, underweight, and short stature [36,37].

According to the relevant literature, scholars have mainly studied the impact of poverty alleviation policies, health shocks, social capital, and social support networks on health poverty vulnerability [17,25]. However, there is a relative lack of research on the relationship between sanitary toilets and health poverty vulnerability, especially for middle-aged and elderly adults in rural Western China. The Ningxia Hui Autonomous Region is located in northwestern China, and its southern mountainous areas are characterized by complex terrain, geographic isolation, and a relative lack of resources. As a result, the region was one of the first contiguous poverty areas in China to receive priority support [38]. Before the full eradication of poverty, Ningxia had a large proportion of poor people, with disease being the main contributor to poverty [9]. Therefore, a comprehensive understanding of the current situation of the vulnerability of middle-aged and elderly adults to health poverty in rural areas of the central and western regions can be targeted to reduce the financial burden on families due to medical expenses caused by physical illnesses, which is highly important for consolidating the fight against poverty, preventing poverty from being caused by illnesses, and returning to poverty.

This study utilizes data from the 2022 Rural Household Health Inquiry Survey to investigate the association between sanitary toilets and health poverty vulnerability among rural western Chinese adults aged 45 years and older. The propensity score matching method is initially employed to examine the net effect of sanitary toilets on this population. Subsequently, robustness tests are performed by altering the explained variable and employing alternative matching techniques. The study then conducted a heterogeneity analysis to divide the research population into distinct subgroups based on gender, age, and income and examined the connection between the use of sanitary toilets and health poverty vulnerability in each subgroup. Finally, a mediation effect analysis was performed to explore the potential mechanisms through which sanitary toilets may influence health poverty vulnerability.

## Methods

### Data sources

The western part of China, the Ningxia Hui Autonomous Region, was the site of a comprehensive health survey conducted in this study in rural areas from June to July 2022. This study used the Rural Household Health Inquiry Survey to gather empirical data and scientific evidence to guide the development of healthcare policies in the region. Since 2009, this study has conducted a baseline survey, followed by five rounds of follow-up surveys in 2011, 2012, 2015, 2019, and 2022. When designing the questionnaire, we sought the opinions of experts in the field of health policy through expert consultation. The feedback from experts was crucial for refining and optimizing the content of the questionnaire. Additionally, before conducting the formal survey, we carried out a presurvey. The presurvey helped us identify issues and make corresponding adjustments to the questionnaire content. After these revisions, the quality of the questionnaire significantly improved. Using a multistage stratified cluster random sampling method, samples were drawn from four counties in Ningxia: Haiyuan, Yanchi, Xiji, and Pengyang. The sampling process comprised three stages. Initially, township administrative villages in each county were classified into three categories—good, medium, and poor—based on their level of economic development. Subsequently, random sampling was conducted to select 40% of the sample villages from each stratum using the random number table method. Finally, systematic sampling was applied to select 20 to 33 households from each village as sample households. The specific sampling details were as follows: Yanchi County had 40 villages, with 33 households selected from each; Haiyuan County contained 76 villages, with 33 households randomly chosen from each; Xiji County comprised 58 villages, with 20 households selected from each; and Pengyang County had 33 villages, with 20 households randomly chosen from each. The survey focused on all permanent household members who had resided in the area for at least six months.

**Fig 1:**
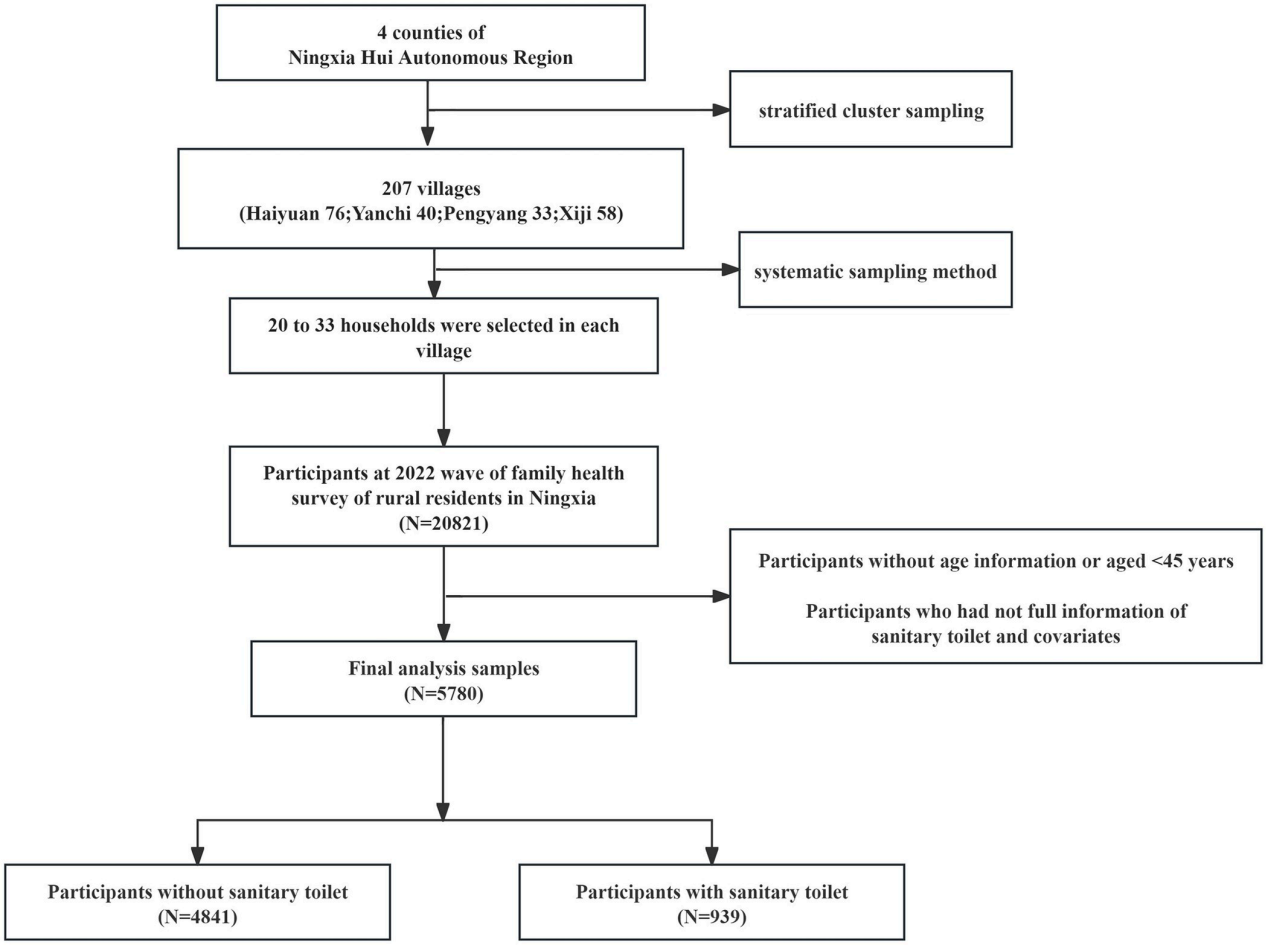
Participant screening process.

Face-to-face questionnaire surveys were conducted by trained graduate students with master’s degrees, which included information on basic personal information, illness and medical consultation status in the past two weeks, hospital treatment situation, chronic disease conditions, health and behavior of family members aged 15 and above, related conditions of married women of childbearing age aged 15–49, status of children under 7 years old, health conditions of residents aged 45 and above, and family economic status. A total of 21,300 questionnaires were distributed in this survey, and 21,300 were returned, of which 20,821 were valid, resulting in an effective response rate of 97.75%. Residents aged 45 years and older were selected for the survey. After excluding samples with missing or invalid values, 5,780 eligible participants were included in the study (Fig 1).

### Model and variables

#### Explained variable (Y): Health poverty vulnerability

Health poverty vulnerability is the probability that, due to health-related risks, a family’s or individual’s welfare decreases below the poverty line, reflecting the risk of falling into poverty in the future. The primary metric used to assess this vulnerability is known as vulnerability as expected poverty (VEP) [39,40]. This method quantifies a household’s susceptibility to health-induced poverty through a three-step process, primarily utilizing the three-stage feasible generalized least squares (FGLS) method [41,42]. The first step involves estimating the income equation using the ordinary least squares (OLS) method, setting the foundation for a detailed analysis of vulnerability.

$$ln\;Y_{it+1}=\beta X_{it}+e_{it}$$ (1)

where *Y*_*it*+1_ refers to the income level of the individual in period *T*+1 and *X*_*it*_ represents the health risk variable that affects income fluctuation. Considering the heterogeneity of different individuals, the squared residuals are used as an approximation of income variance ê2i, and the residual square is used as the dependent variable to construct the regression model of residual square ê2i for individual characteristics:

e^i2=θ×Xi+ηi (2)

The estimated value and the residual estimated value of *Y*_*it*+1_ can be obtained using (1) and (2). Second, the heteroskedasticity matrix is constructed as weights to perform a weighted regression on the logarithm of income (3) and the squared residuals (4):

E^[lnYi∣Xi]=Xiβ^ (3)

V^[lnYi∣Xi]=σ^ei2=Xiθ^ (4)

Finally, the poverty line was used to estimate health poverty vulnerability. The choice of poverty line affects the accuracy of the prediction of health poverty vulnerability. To improve the robustness of our results, we choose two poverty lines: the international poverty line of $1.9 per day and $3.1 per day [43,44]. Given that our research focuses on rural residents aged 45 years and older, the lognormal distribution is particularly relevant for this demographic. Accordingly, the poverty line is logarithmically transformed into Eq (5) to accurately reflect this approach.


v^i=P⏜(lnYi<lnl∣Xi)=φ(lnl−Xiβ^Xiθ^) (5)

*Tobit model*. Health poverty vulnerability is a finite continuous variable; therefore, this study used a Tobit model to analyze the influencing factors. In this case, health poverty vulnerability was used as the dependent variable (V), and demographic characteristics, physical capital, financial capital, social capital, and human capital were used as independent variables (Xj).


$$V=\beta_{0}+\beta_{j}X_{j}+\varepsilon$$ (6)

*Propensity score matching*. This study included adults aged 45 years and older who used sanitary toilets as the treatment group and adults aged 45 years and older who did not use sanitary toilets as the control group. Propensity score matching (PSM) was used to match the covariates between the two groups to control for confounding variables to the greatest extent. Initially, propensity scores for the treatment and control groups were estimated. The logit model was used to calculate propensity scores using the following specific method:

$$Logit(Sanitary\;toilet)=\beta_{0}+\beta_{1}X_{h}+\varepsilon_{h}$$ (7)

Subsequently, a balance check was performed. The balance check aims to determine whether there are still significant differences between the treatment and control groups for each covariate after matching and to assess the changes in the distribution of covariates before and after matching.

#### Mediating variable (M): Livelihood capital

Previous research has demonstrated that household livelihood capital has a significant influence on health poverty vulnerability [20,45,46]. Consequently, this study employs the sustainable livelihood approach (SLA) framework [47]. The principal component of the SLA framework is livelihood capital, which encompasses the diverse resources that households possess to sustain their livelihoods. Health poverty vulnerability was taken as the dependent variable (Y), and sanitary toilet use was taken as the independent variable (X). In conjunction with the questionnaire used in the survey, physical capital (type of housing, type of drinking water, separation of housing and kitchen), financial capital (registered poor household, loans because of illness, total household income), social capital (expenditure on gifts), and human capital (household size, number of chronic disease patients, number of household laborers) were considered mediating variables (M). This study explored the potential pathways through which livelihood capital affects the ability of sanitary toilets to reduce health poverty vulnerability (S1 Table in S4 File).

The mediation effect model was used to analyze the processes and mechanisms of influence between variables. In studying the impact of the independent variable X (sanitary toilets) on the dependent variable Y (health poverty vulnerability), sanitary toilets not only directly affect health poverty vulnerability but also have an indirect impact through the mediating variable M. Therefore, M is considered a mediating variable, and the model describing the relationship between X, M, and Y, which is X→M→Y, is known as the mediation effect model [48,49].

The mediation effect is represented by the coefficient b and the product of a and b, which reflects the extent of the indirect impact of X on Y through M. Given that the independent variable in this study is a categorical variable, the Sobel test was used for mediation effect analysis.


$$Y=cX+e_{1}$$

$$M=aX+e_{2}$$

$$Y=c\prime X+bM+e_{3}$$

#### Explanatory variables (X): Sanitary toilets

In rural areas of China, the use of communal dry toilets is still very common, while flush toilets that the government aims to install in these areas are considered prestigious and desirable [31]. A sanitary toilet refers to a toilet facility that has walls, a roof, and a door and is clean and essentially odorless. The temporary storage or treatment facilities for feces should be leak-proof, with no exposure of feces and no presence of fly larvae [50]. In this survey, participants were asked to identify the type of toilet that they primarily used. The options provided were flush toilets, double-vault funnel toilets, pit toilets, and nonflush dry pit toilets. Flush toilets are usually connected to modern sewage treatment systems and can effectively reduce the risk of pathogen transmission. However, in rural areas of China, double-vault funnel toilets, pit toilets, and nonflush dry-pit toilets were categorized as unsanitary toilets in this study because of insufficient control over pathogen transmission and the difficulty of maintenance and management, leading to poor sanitary conditions. Therefore, in our study, for respondents who use flush toilets, the variable "sanitary toilet" is assigned a value of 1; if the respondent uses double-vault funnel toilets, pit toilets, or nonflush dry pit toilets, the variable "sanitary toilet" is assigned a value of 0. Flush toilets are classified in the Joint Monitoring Programme (JMP) sanitation ladder as safely managed services, which are private improved facilities where fecal waste is safely treated on site or transported and treated elsewhere, along with handwashing facilities with soap and water. Double-vault funnels, pit latrines, and nonflush dry pit toilets are classified as basic services in the Joint Monitoring Programme (JMP) sanitation ladder and are private improved facilities that separate excreta from human contact [51].

#### Control variable (C)

Considering that other variables may simultaneously affect the independent, mediating, and dependent variables, this study used gender, age, marital status, education, occupation, and self-rated health as control variables (Table 1) [20].

**Table 1:** Variable definitions and descriptive statistics.

Variables	Explanation	Code	Mean	SD
Explained variable (Y)	Health poverty vulnerability	Measure with vulnerability as expected poverty (VEP)		
Explanatory variable (X)	Sanitary toilet	Categorical variable, yes = 1 and no = 0	0.1625	0.3689
Control variable (C)	Gender	Categorical variable, male = 1 and female = 2	1.4659	0.4989
	Age	Ordered multicategorical variable, 45–59 years old = 1,60–74 years old = 2,≥75 years old = 3	1.5751	0.6573
	Marital status	Unordered multi categorical variable, unmarried = 1, married = 2, divorced/widowed = 3	2.0787	0.3060
	Education	Ordered multi categorical variable, no schooling = 1, primary school = 2, junior high school = 3, senior high school or above = 4	1.8462	0.8632
	Occupation	Unordered multi categorical variable, farming = 1,work = 2,village cadres and village doctors = 3, small businesses = 4,out of work = 5, else = 6	1.8358	1.5489
	Self-rated health	Ordered multi categorical variable, very good = 1, good = 2, fair = 3, bad = 4, very bad = 5	3.3694	1.0625
Physical capital	Type of housing	Unordered multi categorical variable, brick soil concrete = 1, brick wood2 = 2,full brick = 3, earthen houses and kilns = 4	2.3528	0.7684
	Type of drinking water	Unordered multi categorical variable, 1 = tap water, 2 = cellar water, 3 = well water	1.0616	0.3040
	Separation of housing and kitchen	Categorical variable, yes = 1 and no = 0	0.7304	0.4438
Financial capital	Registered poor household	Categorical variable, yes = 1 and no = 0	0.5209	0.4996
	Loans because of illness	Categorical variable, yes = 1 and no = 0	0.1860	0.3891
	Total household income(log)	Continuous variable (logarithm)	10.1446	0.7802
Social capita	Expenditure on gifts(log)	Continuous variable (logarithm)	7.2508	1.7468
Human capital	Household size	Ordered multicategorical variable,1–3 persons = 1,4–5 persons = 2,≥6 persons = 3	1.5062	0.7179
	Number of chronic disease patients	Continuous variable	1.0128	0.8097
	Number of household labor	Continuous variable	3.0223	1.8839

### Statistical analysis

In our research process, we used EpiData to code the data and thoroughly checked the survey data for missing values prior to the analysis. Statistical analysis was performed using the econometric software SPSS 26.0 and STATA version 17.0. To address mixed-factor issues, we employed the propensity score matching (PSM) method, which includes balance testing and evaluation of the common support hypothesis. PSM and Tobit regression analyses were subsequently performed to determine the association between sanitary toilets and health poverty vulnerability within the matched samples. To ensure the robustness of our findings, we used various propensity score matching methods. Finally, we performed a mediating effect analysis to explore the intermediary pathway linking sanitary toilets to health poverty vulnerability. All the statistical tests in this study were performed at a significance level of *P* < 0.05 using a two-tailed approach.

## Results

### Propensity score matching for the hypothesis of common support

**Fig 2:**
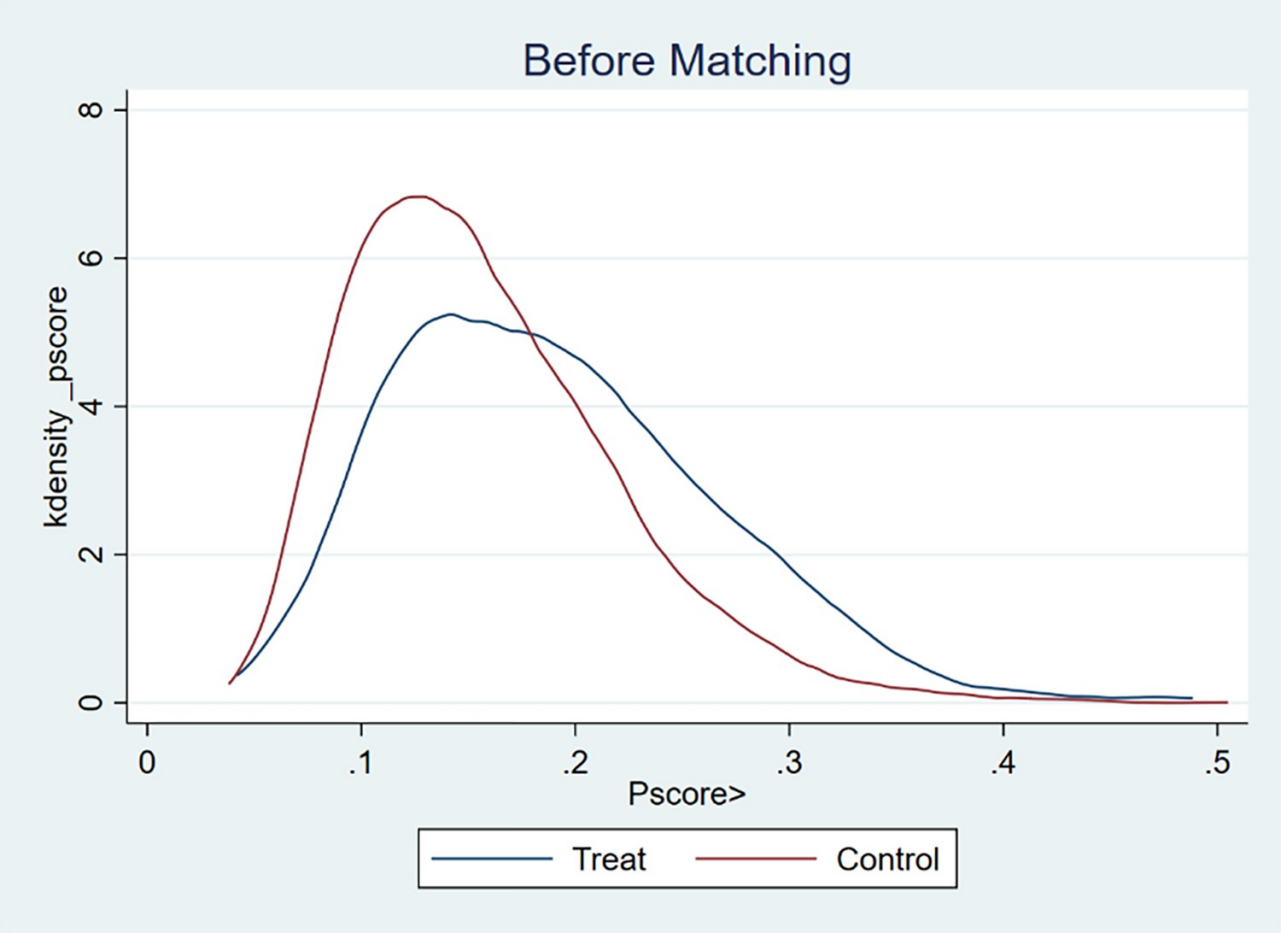
Balance tests before and after matching between the experimental group with access to sanitary toilets and the control group without access to sanitary toilets.

**Fig 3:**
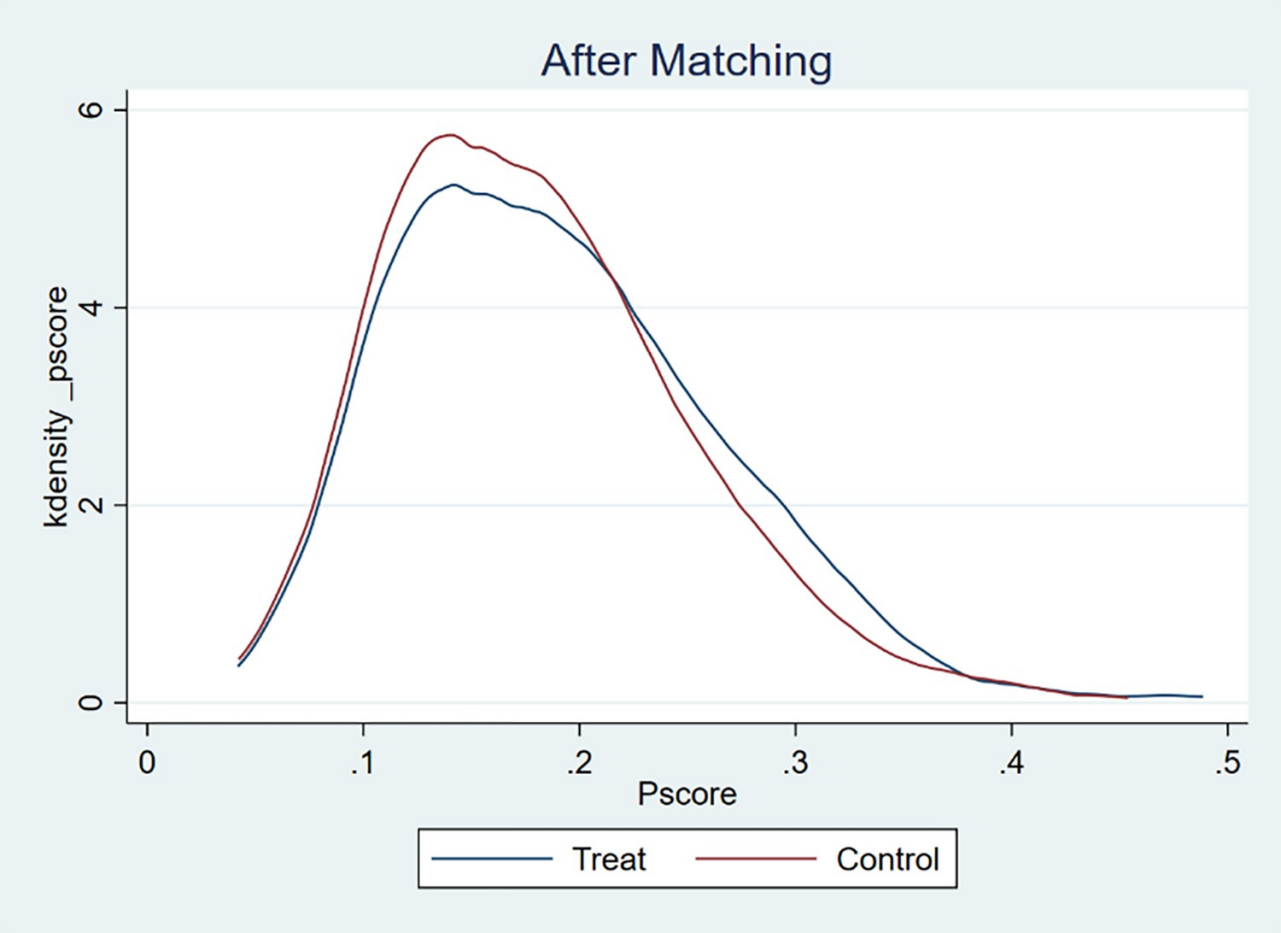
Balance tests before and after matching between the experimental group with access to sanitary toilets and the control group without access to sanitary toilets.

To ensure that the treatment and control groups were similar in terms of individual characteristics, this study used propensity score matching combined with a kernel density function to test the common support hypothesis [52,53]. Figs 2 and 3 show that before matching, there was a significant difference in the kernel density distribution of the propensity scores between the treatment group that used sanitary toilets and the control group that did not use sanitary toilets. After matching, the kernel density distribution curves of the two groups largely overlap, indicating that the matching satisfies the common support assumption.

### Processed the self-selection problem

This study used propensity score matching to explore the association between sanitary toilets and health poverty vulnerability among rural western Chinese adults aged 45 years and older, aiming to reduce the bias introduced by self-selection of the sample. Table 2 shows that after nearest-neighbor matching, the absolute value of the standardized deviation of the matched control variables was less than 10% [54]. The differences between the experimental and control groups were significantly reduced after the matching treatment. In addition, the t test results show that the two matched samples are not significantly different in terms of any control variables. Therefore, the problem of within-sample heterogeneity was partially resolved, and the balance assumption was met.

**Table 2:** Sample balance test of propensity score matching.

Variable (3.1$)	Sample	Mean		Standard deviation%	Deviation reduction %	t test	
		Processing group	Control group			T value	P value
Gender	Before	1.4633	1.4664	-0.6	-1242.2	-0.180	0.858
	After	1.4633	1.4207	8.5		1.860	0.063
Age	Before	1.5911	1.5720	2.9	88.8	0.810	0.416
	After	1.5911	1.5932	-0.3		-0.070	0.945
Marital status	Before	2.0660	2.0812	-5.0	100.0	-1.390	0.165
	After	2.0660	2.0660	0.0		0.000	1.000
Education	Before	1.9840	1.8195	18.7	96.8	5.360	0.000
	After	1.9840	1.9894	-0.6		-0.130	0.898
Occupation	Before	2.0756	1.7893	17.9	99.6	5.200	0.000
	After	2.0756	2.0767	-0.1		-0.010	0.989
Self-rated health	Before	3.2769	3.3873	-10.3	74.0	-2.920	0.004
	After	3.2769	3.2481	2.7		0.580	0.563
Type of housing	Before	2.4462	2.3346	14.9	63.7	4.080	0.000
	After	2.4462	2.4058	5.4		1.160	0.245
Type of drinking water	Before	1.0618	1.0616	0.1	-8508.5	0.020	0.985
	After	1.0618	1.0799	-5.8		-1.220	0.224
Separation of housing and kitchen	Before	0.8072	0.7156	21.6	98.8	5.810	0.000
	After	0.8072	0.8083	-0.3		-0.060	0.953
Registered poor household	Before	0.4271	0.5391	-22.6	86.7	-6.310	0.000
	After	0.4271	0.4121	3.0		0.650	0.513
Loans because of illness	Before	0.1597	0.1911	-8.2	86.4	-2.260	0.024
	After	0.1597	0.1555	1.1		0.250	0.800
Total household income(log)	Before	10.2060	10.1330	9.4	75.2	2.630	0.008
	After	10.2060	10.2240	-2.3		-0.490	0.623
Expenditure on gifts(log)	Before	7.2853	7.2441	2.4	-48.5	0.660	0.509
	After	7.2853	7.2242	3.5		0.760	0.448
Household size	Before	1.4036	1.5261	-17.5	88.7	-4.790	0.000
	After	1.4036	1.4175	-2.0		-0.450	0.653
Number of chronic disease patients	Before	1.0043	1.0145	-1.3	-87.9	-0.350	0.724
	After	1.0043	1.0234	-2.4		-0.510	0.612
Number of household labor	Before	2.7966	3.0661	-14.3	86.2	-4.020	0.000
	After	2.7966	2.8339	-2.0		-0.440	0.663

### Comprehensive evaluation of the association between sanitary toilets and health poverty vulnerability

The outcomes of the Tobit and propensity score matching regressions, presented in columns (1)–(4) of Table 3, show the association between sanitary toilets and health poverty vulnerability among rural western Chinese adults aged 45 years and above. Column (2) of the Tobit regression reveals that the utilization of sanitary toilets is associated with a statistically significant reduction in health poverty vulnerability (β = -0.0340, *P*<0.05). Furthermore, the results in column (4) of the propensity score-matched regression demonstrate that the use of sanitary toilets is a significant factor in reducing the vulnerability of middle-aged and elderly adults to health poverty in rural areas, with the estimated coefficient being statistically significant at the 10% level.

**Table 3:** Association between sanitary toilets and health poverty vulnerability among rural western Chinese adults aged 45 years and older.

Variable (3.1$)	(1)	(2)	(3)	(4)
Sanitary toilet	-0.1368***	-0.0340**	-0.0329	-0.0342*
	(0.0206)	(0.0135)	(0.0261)	(0.0180)
Gender		-0.0120		-0.0024
		(0.0107)		(0.0195)
Age		-0.0091		-0.0138
		(0.0093)		(0.0168)
Marital status		-0.1079***		-0.0825**
		(0.0173)		(0.0325)
Education		-0.0055		-0.0153
		(0.0064)		(0.0115)
Occupation		-0.0054		-0.0020
		(0.0035)		(0.0059)
Self-rated health		0.0134***		0.0090
		(0.0051)		(0.0092)
Type of housing		-0.0064		-0.0150
		(0.0065)		(0.0119)
Type of drinking water		0.0115		0.0177
		(0.0167)		(0.0292)
Separation of housing and kitchen		-0.0011		0.0009
		(0.0114)		(0.0232)
Registered poor household		0.0267***		0.0359*
		(0.0102)		(0.0189)
Loans because of illness		0.0128		0.0473*
		(0.0132)		(0.0258)
Total household income(log)		-0.5583***		-0.4795***
		(0.0083)		(0.0145)
Expenditure on gifts(log)		-0.0299***		-0.0277***
		(0.0033)		(0.0061)
Household size		0.3259***		0.2711***
		(0.0113)		(0.0215)
Number of chronic disease patients		-0.0091		-0.0152
		(0.0067)		(0.0121)
Number of household labor		0.0448***		0.0393***
		(0.0044)		(0.0082)
N	5780	5780	1657	1657
R^2^	0.0043	0.4983	0.0006	0.4616
Prob>F	<0.001	<0.001	<0.001	<0.001

***P < 0.01

**P < 0.05

*P < 0.1.Standard errors in parentheses.

The Tobit regression results presented in column (2) reveal that lower self-assessed health status, registration as poor, larger household size, and increased number of household laborers are all variables that exacerbate health poverty vulnerability among middle-aged and elderly adults living in rural areas. These variables have statistically significant coefficients at the 1% level. On the other hand, marital status, higher total household income, and higher expenditures on gift money are factors that mitigate the risk of health poverty for middle-aged and older rural adults. These factors exhibit statistically significant coefficients at the 1% level.

### Robustness test based on the explained variable

This study assessed the stability of the regression findings by substituting the explained variables. To achieve this goal, health poverty vulnerability was replaced with a poverty line of $3.1, while a lower poverty line of $1.9 was used for robustness testing purposes. The findings from the Tobit regression model with control variables are presented in columns (1) and (2) of Table 4, whereas the PSM regression analysis with control variables is shown in columns (3) and (4) of the same table. The results of both the Tobit regression column (2) and PSM regression column (4) indicate that the use of sanitary toilets was significantly associated with decreased health poverty vulnerability in adults over 45 years of age, with the estimated coefficients being statistically significant at the 10% level.

**Table 4:** Robustness tests based on the explained health poverty vulnerability at the $1.9 poverty line.

Variable(1.9$)	(1)	(2)	(3)	(4)
Sanitary toilet	-0.0833***	-0.0209*	-0.0195	-0.0232*
	(0.0141)	(0.0108)	(0.0175)	(0.0140)
Control variable	Yes	Yes	Yes	Yes
N	5780	5780	1657	1657
R^2^	0.006	0.5848	0.001	0.5757
Prob>F	<0.001	<0.001	<0.001	<0.001

***P < 0.01

**P < 0.05

*P < 0.*1*.Standard errors in parentheses.

### Robustness test based on the matching approach

To ensure the robustness and reliability of the research methodology, different propensity score matching methods were used in this study for robustness analysis, including radius matching, kernel matching, local linear matching, and Mahalanobis distance matching [55,56]. Table 5 shows that, with the exception of the Mahalanobis distance matching method, all three matching methods indicate that the use of sanitary toilets was significantly associated with decreased health poverty vulnerability in adults over 45 years of age, with estimated coefficients significant at the 10% level.

**Table 5:** Robustness test based on the matching approach.

Variable	Radius matching		Nuclear matching		Local linear matching		Mahalanobis distance matching	
	1.9$	3.1$	1.9$	3.1$	1.9$	3.1$	1.9$	3.1$
Sanitary toilet	-0.0232*	-0.0342*	-0.0209*	-0.0340**	-0.0235*	-0.0338*	0.0026	-0.0059
	(0.0140)	(0.0180)	(0.0108)	(0.0135)	(0.0140)	(0.0180)	(0.0111)	(0.0140)
Control variable	Yes	Yes	Yes	Yes	Yes	Yes	Yes	Yes
N	1657	1657	5780	5780	1656	1656	3238	3238
R^2^	0.5757	0.4616	0.5848	0.4983	0.5757	0.4615	0.6253	0.5111
Prob>F	<0.001	<0.001	<0.001	<0.001	<0.001	<0.001	<0.001	<0.001

***P < 0.01

**P < 0.05

*P < 0.*1*.Standard errors in parentheses.

### Heterogeneity

Table 6 analyses the association between sanitary toilets and health poverty vulnerability among rural western Chinese adults aged 45 years and above stratified by gender, age, and total household income. The results showed that from a gender perspective, sanitary toilets were significantly associated with decreased health poverty vulnerability among males (β = -0.0375, *P*<0.05), but the effect on females was not significant (β = -0.0300, *P*>0.05). In terms of age, sanitary toilets were significantly associated with decreased health poverty vulnerability among those aged 60–74 years (β = -0.0476, *P*<0.05), but the effect was not significant for those aged 45–59 years (β = -0.0171, *P*>0.05) or 75 years or older (β = -0.0641, *P*>0.05). In terms of total household income, sanitary toilets were significantly associated with decreased health poverty vulnerability in middle-income households (β = -0.0590, *P*<0.01), but the effect was not significant in high-income households (β = 0.0086, *P*>0.05).

**Table 6:** Heterogeneity analysis of gender, age, and income.

Variable(3.1$)	Male	Female	45–59 age group	60–74 age group	≥75 age group	Low–income	Middle–income	High–income
Sanitary toilet	-0.0375**	-0.0300	-0.0171	-0.0476**	-0.0641	-0.0549*	-0.0590***	0.0086
	(0.0186)	(0.0197)	(0.0184)	(0.0218)	(0.0448)	(0.0297)	(0.0178)	(0.0138)
Gender			-0.0138	-0.0122	0.0111	-0.0440*	0.0115	-0.0159
			(0.0143)	(0.0176)	(0.0367)	(0.0242)	(0.0136)	(0.0111)
Age	-0.0055	-0.0144				-0.0686***	-0.0224*	0.0265**
	(0.0125)	(0.0141)				(0.0203)	(0.0121)	(0.0104)
Marital status	-0.1000***	-0.1147***	-0.0669**	-0.1201***	-0.2301***	-0.4449***	0.0270	-0.0240
	(0.0248)	(0.0245)	(0.0272)	(0.0291)	(0.0399)	(0.0334)	(0.0234)	(0.0217)
Education	0.0001	-0.0136	-0.0214***	0.0179*	-0.0481	-0.0207	-0.0007	-0.0175***
	(0.0080)	(0.0105)	(0.0082)	(0.0105)	(0.0292)	(0.0153)	(0.0082)	(0.0063)
Occupation	-0.0085*	-0.0019	-0.0039	-0.0053	-0.0205	-0.0048	0.0021	-0.0032
	(0.0050)	(0.0051)	(0.0063)	(0.0051)	(0.0091)	(0.0074)	(0.0046)	(0.0039)
Self-rated health	0.0169**	0.0090	0.0093	0.0148*	0.0305*	0.0298**	0.0100	0.0006
	(0.0070)	(0.0075)	(0.0068)	(0.0084)	(0.0183)	(0.0125)	(0.0064)	(0.0052)
Type of housing	-0.0051	-0.0073	0.0075	-0.0186*	-0.0227	0.0070	-0.0042	-0.0145**
	(0.0089)	(0.0094)	(0.0089)	(0.0103)	(0.0219)	(0.0140)	(0.0080)	(0.0071)
Type of drinking water	0.0099	0.0119	0.0188	0.0100	-0.0941	0.0655	-0.0101	-0.0156
	(0.0231)	(0.0241)	(0.0213)	(0.0286)	(0.0685)	(0.0407)	(0.0215)	(0.0175)
Separation of housing and kitchen	0.0029	-0.0056	0.0128	-0.0084	-0.0319	0.0058	0.0009	0.0426***
	(0.0158)	(0.0166)	(0.0155)	(0.0188)	(0.0392)	(0.0266)	(0.0147)	(0.0119)
Registered poor household	0.0307**	0.0227	0.0339**	0.0099	0.0614*	0.0272	0.0506***	0.0025
	(0.0141)	(0.0148)	(0.0137)	(0.0168)	(0.0347)	(0.0231)	(0.0128)	(0.0108)
Loans because of illness	0.0024	0.0245	-0.0066	0.0500**	-0.0146	0.0476	-0.0209	0.0035
	(0.0182)	(0.0193)	(0.0175)	(0.0219)	(0.0500)	(0.0335)	(0.0174)	(0.0129)
Total household income(log)	-0.5625***	-0.5528***	-0.5329***	-0.5697***	-0.6846***			
	(0.0113)	(0.0122)	(0.0111)	(0.0136)	(0.0301)			
Expenditure on gifts(log)	-0.0317***	-0.0285***	-0.0349***	-0.0325***	-0.0042	-0.0180**	-0.0030	-0.0185***
	(0.0045)	(0.0048)	(0.0047)	(0.0052)	(0.0114)	(0.0085)	(0.0041)	(0.0034)
Household size	0.3358***	0.3144***	0.2819***	0.3664***	0.4715***	2.8481	0.6578***	0.1676***
	(0.0154)	(0.0166)	(0.0147)	(0.0199)	(0.0422)	(34.6815)	(0.0165)	(0.0106)
Number of chronic disease patients	-0.0110	-0.0066	-0.0006	-0.0123	-0.0279	0.0272*	0.0020	-0.0021
	(0.0090)	(0.0099)	(0.0087)	(0.0113)	(0.0239)	(0.0162)	(0.0085)	(0.0067)
Number of household labor	0.0420***	0.0476***	0.0506***	0.0408***	0.0335**	0.0850***	0.0604***	0.0176***
	(0.0061)	(0.0065)	(0.0064)	(0.0071)	(0.0138)	(0.0102)	(0.0064)	(0.0045)
N	3087	2693	2998	2240	542	1927	1924	1,929
R2	0.5035	0.4925	0.5171	0.4825	0.5333	0.5323	0.8895	1.2044
Prob>F	<0.001	<0.001	<0.001	<0.001	<0.001	<0.001	<0.001	<0.001

***P < 0.01

**P < 0.05

*P < 0.1.Standard errors in parentheses.

### Intermediary mechanism analysis

Table 7 shows that health poverty vulnerability was used as the dependent variable, sanitary toilets were used as the independent variable, and the dimensions of physical, financial, social, and human capital were used as the mediating variables, while demographic variables were included in the analysis of the mediated effects model as control variables. The Sobel test for mediating effects showed that registered poor households (a*b = -0.0081, *P*<0.01, or 8.14% of the total effect), total household income (a*b = -0.0233, *P*<0.05, or 23.46% of the total effect), household size (a*b = -0.0181, *P*<0.01, or 18.27% of the total effect), and number of laborers in the household (a*b = -0.0107, *P*<0.01, 10.82% of the total effect) all negatively predicted health poverty vulnerability.

**Table 7:** Mediating effect test of health poverty vulnerability related to sanitary toilets.

Variable(3.1$)	a coefficient	b coefficient	Indirect effect (a*b)	Direct effect	Total effect	Proportion of total effect that is mediated
Type of housing	0.1132***	-0.0151*	-0.0017*	-0.0974***	-0.0991***	0.0173
	(0.0275)	(0.0080)	(0.0010)	(0.0168)	(0.0168)	
Type of drinking water	0.0044	0.0323	0.0001	-0.0993***	-0.0991***	-0.0014
	(0.0109)	(0.0203)	(0.0004)	(0.0168)	(0.0168)	
Separation of housing and kitchen	0.0835***	0.0170	0.0014	-0.1006***	-0.0991***	-0.0143
	(0.0158)	(0.0140)	(0.0012)	(0.0169)	(0.0168)	
Registered poor household	-0.0927***	0.0870***	-0.0081***	-0.0911***	-0.0991***	0.0814
	(0.0177)	(0.0125)	(0.0019)	(0.0168)	(0.0168)	
loans because of illness	-0.0150	-0.0653***	0.0010	-0.1001***	-0.0991***	-0.0099
	(0.0137)	(0.0162)	(0.0009)	(0.0168)	(0.0168)	
Total household income(log)	0.0644**	-0.3613***	-0.0233**	-0.0759***	-0.0991***	0.2346
	(0.0269)	(0.0067)	(0.0097)	(0.0138)	(0.0168)	
Expenditure on gifts(log)	0.0475	-0.0866***	-0.0041	-0.0950***	-0.0991***	0.0415
	(0.0600)	(0.0035)	(0.0052)	(0.0160)	(0.0168)	
Household size	-0.1155***	0.1567***	-0.0181***	-0.0810***	-0.0991***	0.1827
	(0.0252)	(0.0085)	(0.0041)	(0.0164)	(0.0168)	
Number of chronic disease patients	-0.0041	-0.0074	0.0000	-0.0992***	-0.0991***	-0.0003
	(0.0270)	(0.0082)	(0.0002)	(0.0168)	(0.0168)	
Number of household labor	-0.2299***	0.0467***	-0.0107***	-0.0884***	-0.0991***	0.1082
	(0.0634)	(0.0034)	(0.0031)	(0.0166)	(0.0168)	

***P < 0.01

**P < 0.05

*P < 0.1.Standard errors in parentheses.

## Discussion

The ’Toilet Revolution’ became a buzzword in China. Against this backdrop, this study used data from the 2022 Ningxia Rural Household Health Inquiry Survey to explore the association between sanitary toilets and health poverty vulnerability among rural western Chinese adults aged 45 years and older. The study results indicate that the use of sanitary toilets was significantly associated with decreased health poverty vulnerability in adults over 45 years of age. This outcome may be attributed to the effective prevention of fecal contamination of food and water sources by sanitary toilets, thereby reducing the incidence of gastrointestinal infectious diseases [57,58]. Based on these findings, this study recommends that the government provide financial support for constructing sanitary toilets, especially in rural and economically disadvantaged areas. Furthermore, it emphasizes the need for increased investment in infrastructure development to aid this vulnerable group further.

This study highlights the association between sanitary toilets and health poverty vulnerability, noting significant disparities across sex, age, and income groups. A targeted and orderly approach should be adopted to advance the Toilet Revolution in China, with categorized promotion [59]. Heterogeneity analysis revealed that sanitary toilets were significantly associated with decreased health poverty vulnerability among males but had no significant association with the health poverty vulnerability of females. Because women enter perimenopause and menopause after the age of 45, although sanitary toilets reduce their exposure to gastrointestinal pathogens with changes in hormone levels, women in this age group may face an increased risk of chronic diseases such as osteoporosis and cardiovascular diseases [60]. Therefore, sanitary toilets were significantly more strongly associated with decreased health poverty vulnerability in women than in men. In terms of age groups, sanitary toilets were significantly associated with decreased health poverty vulnerability in the population aged 60–74 years. This may be related to the gradual weakening of the immune system with age, which increases the susceptibility of this age group to pathogens [61]. Sanitary toilets play a key role in preventing the spread of pathogens [62], thus providing more effective health protection for people. Furthermore, the study revealed that the impact of sanitary toilets is more substantial among middle-income groups. Limited financial resources make these groups more dependent on public infrastructure and services to meet their hygiene needs. The use of sanitary toilets can significantly improve hygiene practices, thereby effectively reducing health poverty vulnerability in these income groups.

Mediating effect analysis indicates that total household income, household size, the number of household laborers, and whether the household is registered as impoverished play significant mediating roles between sanitary toilets and health poverty vulnerability. Total household income plays a pivotal role in significantly alleviating the vulnerability of the rural elderly to health poverty, as a higher income level enables individuals to accumulate wealth and effectively manage health-related risks [63]. Moreover, individuals with higher incomes typically possess better health literacy and pay more attention to their health status [64]. Household size played a significant mediating role in alleviating health poverty vulnerability. Larger families tend to have broader social support networks, which are crucial for providing emotional, financial, and daily life support [65]. Such support contributes to the promotion of the physical and mental health of the rural elderly, thereby reducing vulnerability to health poverty. The number of household laborers is also an important mediating factor in alleviating health poverty vulnerability. A greater number of laborers within a family implies that more individuals are engaged in agricultural work, manual labor, and income-generating activities. This diversification of income sources is crucial for mitigating the financial burdens caused by illness. Additionally, being registered as an impoverished household was a significant mediating pathway. By integrating poverty subsidies with medical insurance policies, medical costs can be reduced, and the coverage of medical services can be expanded [66], thereby reducing vulnerability to health poverty.

This study has certain limitations. First, this is a cross-sectional study that can explore the association between sanitary toilets and health poverty vulnerability, but the causal relationship between sanitary toilets and health poverty vulnerability cannot be inferred. Second, this was a retrospective survey, and the information of the respondents was self-reported. Although the investigators were all graduate students and a one-on-one questioning method was used to collect the information of the respondents, recall bias could not be completely avoided. Additionally, this study primarily focused on older adults in rural areas of specific provinces in Western China; hence, the research findings may not be readily generalizable to other regions. Future studies should consider using panel data that cover broader demographic data to provide more comprehensive research.

## Conclusion

This study evaluated the association between sanitary toilets and health poverty vulnerability among rural western Chinese adults aged 45 years and older by utilizing data from the 2022 Ningxia Rural Household Health Inquiry Survey. The study revealed that sanitary toilets were significantly associated with decreased health poverty vulnerability in men over 45 years of age. Heterogeneity analysis revealed that this effect was particularly pronounced among males, individuals aged 60 to 74 years, and families with middle incomes. Additionally, the mediation effect analysis revealed that livelihood capital plays a mediating role in the relationship between sanitary toilets and health poverty vulnerability; the mediation effect size decreased in the following order: total household income, household size, number of household laborers, and registered poor households.

## Policy implications

This study indicated that the use of sanitary toilets was significantly associated with decreased health poverty vulnerability in adults over 45 years of age. As such, policymakers should prioritize the construction of sanitary toilets and other hygiene facilities in rural areas, particularly in economically underdeveloped regions, to ensure that residents can enjoy basic sanitary conditions. Additionally, it is important to emphasize the enhancement of the livelihood capital of rural households, such as through the provision of employment training, the expansion of sources of income, and the increase in targeted support for poor households, as a foundation for improving health levels. Finally, raising awareness among rural residents about the importance of sanitary toilets, strengthening health education, and specifically highlighting the benefits to the health of middle-aged and elderly people can enhance initiative and proactivity in toilet renovation.

## Supporting information

S1 File Survey questionnaire. (PDF)

S2 File Multistage random sampling counties. (XLSX)

S3 File Multistage random sampling counties. (PDF)

S4 File Supplementary table. (DOCX)
