# Correction: Association between sanitary toilets and health poverty vulnerability among rural western Chinese adults aged 45 years and older: A cross-sectional study

**DOI:** 10.1371/journal.pone.0311844

**Published:** 2025-01-31

**Authors:** The PLOS ONE Editors

## Notice of republication

This article was republished on September 27, 2024, to address an issue identified post-publication. Please download this article again to view the correct version.

The article’s Data Availability statement is updated to: Data for this study cannot be shared publicly due to confidentiality concerns. Data are available from the School of Public Health, Ningxia Medical University (contact 230240130047@nxmu.edu.cn) for researchers who meet the criteria for access to confidential data.
