# Supplementary material for: Association between sanitary toilets and health poverty vulnerability among rural western Chinese adults aged 45 years and older: A cross-sectional study
Source: PLoS One. 2024 Sep 20;19(9):e0308688. doi: 10.1371/journal.pone.0308688 (PMC11414992; doi:10.1371/journal.pone.0308688)
Supplement: S3 File — (PDF) [file pone.0308688.s003.pdf]

List of Haiyuan County (17 villages and 79 villages)				
th	villages and towns	village	Village doctor's name	contact number
	The sea town	Li Zhuang village		
		Wang Jing village		
		Chengguan village		
		Xiguan village		
		Liu yuan village		
		Bao zi village		
		Shuiwa North Village		
	River Town (Black City)	Hongcheng village		
		Tangbao village		
		Four camp village		
		amaranth hemp village		
		Dai shop village		
	Gao Ya township	Gao Ya village		
		United village		
		Perfume village		
		Red bank village		
	Guan bridge township	Ma Wan village		
		Wang Wan village		
		Luo Shan village		
		Zhang Wan village		
		Shuangjing		

		village		
	Gan urban and rural areas	Shi Jing village		
		Yan Wan village		
		Wu canal village		
	Seven camp township	Gao Ya village		
		Zhang fort village		
		Yang fort village		
		Marburg village		
		Under the set of village		
		Five camp village		
	Shi dian township	Mi bay village		
		Cangwan village		
	Jia Tang township	After pond village		
		Wang Tang village		
		Jia Tang village		
		Ma Ying village		

Haiyuan County		Fort village		
		Deux-Rivières		
	Li Jun township	Bao Bao village		
		Yongfeng village		
		Red star village		
		Cai Xiang village		
		Li Jun village		
	Red sheep township	Jianguo village		
		Zhang Yuan village		
		Anbao village		
		Forward village		
		ShuChuan village		
		Liu set village		
		Shitang village		
	Guan zhuang township	Kiln son village		
		Guan zhuang village		
		Waterlogging village		
	Zheng Qi township	West along the village		
		In ping village		
		Zheng Qi village		
		Wu Wan village		
		Old crow village		
		Nanshan		

		village		
		After the mountain village		
		Sa fort village		
	Cao wa township	DeLie village		
		Nitro ditch village		
	Li Wang township	Erdao village		
		Ma Lian village		
		Red scroll village		
		Li orchard village		
		Yang mountain village		
	Xi'an township	Fan tai village		
		Xi'an village		
		Hu Wan village		
		Garden village		
		Tuan zhuang village		
		TongTong village		

	Tree township	Two hundred villages		
		Red well village		
		Xinzhuang village		
	Nine color township	Ma circle village		
		Yuan set village		

List of households in Yanchi County (8 towns and 40 villages)				
**Yanchi County**	**villages and towns**	**village**	**Village doctor's name**	**contact number**
	Hemp yellow mountain	Dissected Xi'an village		
		Houwa village		
		Bao yuan village		
		Huangyangling Village		
		Songji water village		
	Big water pit	New village		
		Sha cao bay village		
		Hongjingzi village		
		Place banquet well village		
		Dongfeng village		
		Ma Fang village		
	green hill	Wangsitan Village		
		GuFengZhuang Village		
		Yingpan village		
	Flower horse pool town	Great Wall village		
		Tong Ji circle village		
		LiuYangBao village		
		Honggou Liang Village		
		Guo Jigou village		
		Sidun son village		
		Barcha Liang village		
		Yu Xing village		
		YingDe village		

	High sand nest	Gaosha Wo village		
		Shi Ji circle village		
		Two step pit village		
		Li Zhuangzi village		
	Wang Lejing	Wang Lejing village		
		Guan beach village		
		Liu Siqu village		
		Wolf hole ditch village Donggou natural village		
		Zeng Ji bank village		
	Feng Ji ditch	Rain strong village		
		Back to LiuZhuang Village		
		Storm spring village		
	Hui'an fort township	Du Jigou village		
		Wolf cloth palm village		
		Meng city village		

		Dam village		
		Hui’an fort village		

Pengyang County household list (10 towns and 33 villages)				
	**villages and towns**	**village**	**Village doctor's name**	**contact number**
**Pengyang County**	Honghe town	Hanbao village		
		He yuan village		
		On the king village		
	Chengyang township	Great Wall village		
		Chengyang village		
		Hanzhai village		
	Ancient town	Ancient city village		
		Gao Zhuang village		
		Nay river village		
		Yang fang village		
	Luo wa township	Luo wa village		
		Village village		
	The fork township	The fork village		
		Pass village		
	Wang wa town	Villa village		
		Li Cha village		
		Wang wa village		
		North wa village		
		Road village village		
	[illegible]	Small [illegible]		

	fork township	fork village		
		Elm village		
	Grass temple township	Zhang street village		
		Zhouzhuang village		
		Mi yuan village		
	New set township	Mao fort village		
		Zhang Hua village		
		Yao river village		
		White river village		
		Xiamawa village		
	Bai Yang town	ShuangMo village		
		Yao river village		
		Ren Wan village		
		Slope village		

List of Xiji County (18 villages and 58 villages)				
	villages and towns	village	Village doctor's name	contact number
Xiji County	Bai Ya township	Bai Ya village		
		Small slope village		
		Yu set of village		
	Will Taiwan township	Deep fork village		
		Mao ditch village		
		Mou rong village		
		Xiping village		
	Ma Jian township	Ma Jian village		
		Big bay village		
		Pang Wan village		
	Ma Lian township	Badai ditch village		
		Makigou village		
		New fort village		
	Flat peak township	Chen tan village		
		Ge cha village		
		Zhang Wu village		
	Subao township	Longchuan village		
		Meng Wan village		
		Zhang Liu village		
		HongZhuang g village		
		Nitrate		

	Nihe township	river village		
		Longburg village		
		Red spring village		
	Wang Min township	Small fork village		
		XueYang village		
		SanCha village		
	Under the fort township	Beizhuang village		
		Yu mu village		
	New camp township	Xinying village		
		GanGou village		
		HongZhuang village		
	Partial urban and rural	Partial city village		
		Slot brain village		
		Dazhuang village		
	Jade bridge township	Jade bridge village		
		Xiabaozi village		

		Big fork village		
	Xinglong township	Xinglong village		
		Yao Du village		
		Wang ditch village		
	Xingping township	Wang Wan village		
		Gao Ya village		
		Yang cha village		
	Huoshizhai Township	Dazhuang village		
		Small chuan village		
		Stone mountain village		
	Strong township	Datan village		
		Gou new village		
		Sheep road village		
		High village		
		Spring bay village		
	ShaGou township	ShaGou village		
		Gu ditch village		
		Donggou village		
	Shi zi township	Nantai village		
		Beitai village		
		Defend the village		
		Li Hai village		
