# Supplementary material for: Association between sanitary toilets and health poverty vulnerability among rural western Chinese adults aged 45 years and older: A cross-sectional study
Source: PLoS One. 2024 Sep 20;19(9):e0308688. doi: 10.1371/journal.pone.0308688 (PMC11414992; doi:10.1371/journal.pone.0308688)
Supplement: S4 File — (DOCX) [file pone.0308688.s004.docx]

**Supplementary Table 1 The definition of main variables**

| Variables | Explanation | Definition |
| --- | --- | --- |
| Explained variable (Y) | Health poverty vulnerability |  |
| Explanatory variable (X) | Sanitary toilet | Categorical variable, yes = 1 and no = 0 |
| Control variable (C) | Gender | Categorical variable, male = 1 and female = 2 |
|  | Age | Ordered multi-categorical variable ,45-59 years old=1,60-74 years old=2,≥75 years old=3 |
|  | Marital status | Unordered multi categorical variable , unmarried =1, married =2 , divorced/widowed =3 |
|  | Education | Ordered multi categorical variable , no schooling =1, primary school =2, junior high school =3, senior high school or above =4 |
|  | Occupation | Unordered multi categorical variable, farming=1,work=2,village cadres and village doctors=3, small businesses=4,out of work=5, else=6 |
|  | Self-rated health | Ordered multi categorical variable, very good=1，good=2，fair =3，bad=4，very bad=5 |
| Material capital | Type of housing | Your family's housing type:Unordered multi categorical variable , brick soil concrete =1, brick wood2=2,full brick=3 ,earthen houses and kilns=4 |
|  | Type of drinking water | The main type of drinking water in your home:Unordered multi categorical variable ,1 = tap water, 2 = cellar water, 3= well water |
|  | Separation of housing and kitchen | Is the housing separate from the kitchen?:Categorical variable, yes = 1 and no = 0,Categorical variable, yes = 1 and no = 0 |
| financial capital | Registered poor household | Is your family classified as a documented poor or low-income household?:Categorical variable, yes = 1 and no = 0 |
|  | Loans because of illness | In the past 6 months, has your family borrowed money from anyone for medical care?:Categorical variable, yes = 1 and no = 0 |
|  | Total household income | Family income in the past year (dollars):Continuous variable (logarithm) |
| Social capita | Gift expenditure(log) | Gift expenses such as dowry, New Year's gifts, wedding gifts, funeral expenses, gifts for relatives and friends:Continuous variable (logarithm) |
| Human capital | Household size | Number of Usual Residents (including Usual Household Population) in the last 6 months:Continuous variable |
|  | Number of chronic disease patients | The number of household members who suffer from chronic disease (person):Continuous variable |
|  | Number of household labor | The number of household members who earn income (person):Continuous variable |
